# Allicin protects against LPS-induced cardiomyocyte injury by activating Nrf2-HO-1 and inhibiting NLRP3 pathways

**DOI:** 10.1186/s12872-023-03442-1

**Published:** 2023-08-18

**Authors:** Fangyuan Sun, Kailiang Xu, Jiayi Zhou, Wei Zhang, Guihe Duan, Ming Lei

**Affiliations:** 1 Trauma emergency center, The Seventh People’s Hospital of Shanghai University of Traditional Chinese Medicine, No.358, Datong Road, Pudong New Area, Shanghai, 200137 China; 2 Department of Critical Care Medicine, The Seventh People’s Hospital of Shanghai University of TCM, Shanghai, 200137 China; 3 Department of Critical Care Medicine, The Shache County People’s Hospital of Xinjiang Kashgar Prefecture, Xinjiang, 844710 China

**Keywords:** Cardiomyocytes, Lipopolysaccharide, Allicin, Apoptosis, Inflammation, NLR family pyrin domain containing 3, Nuclear factor erythroid 2-related factor 2

## Abstract

**Background:**

Allicin is a bioactive compound with potent antioxidative activity and plays a protective effect in myocardial damage and fibrosis. The role and mechanism of Allicin in septic cardiomyopathy are unclear. In this study, we investigated the effects and underlying mechanisms of Allicin on lipopolysaccharide (LPS) induced injury in H9c2 cardiomyocytes.

**Methods:**

H9c2 cardiomyocyte cells were pretreated with Allicin (0, 25, 50, and 100 µM) for 2 h, followed by incubation with LPS (10 µg/mL) for 24 h at 37 °C. Cell viability (cell counting kit-8 [CCK-8]), apoptosis (TUNEL staining), oxidative stress (malondialdehyde [MDA] and superoxide dismutase [SOD]), and cytokines release (Interleukin beta [IL-β], Interleukin 6 [IL-6], and tumor necrosis factor-alpha [TNF-α]) were determined. The mRNA and protein expression of nuclear factor erythroid 2-related factor 2 (Nrf2), heme oxygenase-1 (HO-1), and NLR family pyrin domain containing 3 (NLRP3) signaling pathway molecules were quantified by real-time quantitative PCR (RT-qPCR) and western blot, respectively.

**Results:**

Allicin had no effect on H9c2 cell viability but attenuated LPS-induced injury, with increased cell viability, reduction in inflammatory cytokines release, apoptosis, reduced MDA, and increased SOD (P < 0.05). Additionally, Allicin increased Nrf2 and cellular HO-1 expressions in LPS-treated H9c2 cells. Moreover, Allicin modulated the NLRP3 inflammasome, increased the cleaved caspase-1 (p10) protein, and attenuated the LPS-induced increase in NLRP3, pro-IL-1β, and IL-1β proteins. Silencing of Nrf2 by siRNA (siNrf2) significantly attenuated Allicin-induced increase in cell viability and HO-1 and decrease in NLRP3 protein in LPS-stimulated H9c2 cells.

**Conclusions:**

Allicin protects cardiomyocytes against LPS‑induced injury through activation of Nrf2/HO-1 and inhibition of NLRP3 signaling pathways.

**Supplementary Information:**

The online version contains supplementary material available at 10.1186/s12872-023-03442-1.

## Background

Sepsis is a systemic inflammatory disorder with the presence of bacteria, viral or fungal infections [1], and pose a challenge to the clinician with high incidence and mortality rates [2]. A new scientific report states that there were 48.9 million cases and 11 million deaths globally due to sepsis, which made up over 20% of all fatalities worldwide [3]. However, the heart is one of the vital organs susceptible to sepsis-induced injury, and myocardial dysfunction is one severe complication induced by excess free radical production [4]. Lipopolysaccharide (LPS) is a component in the outer membrane of bacteria and can induce septic myocardial injury through a complex pathophysiological process [5]. However, it is still a major challenge to alleviate sepsis-induced myocardial injury. Moreover, some recent reports have shown that herbal medicines could improve cardiac function and reduce myocardial enzyme levels in septic cardiomyopathy [6, 7]. Therefore, it urgently needs more effective drugs for protection against myocardial injury in septic cardiomyopathy.

An oxidative stress response is considered to be a sign of sepsis-induced cardiotoxicity, and enhanced endogenous antioxidant activity has prominent cardioprotective effects [8]. Oxidative stress can activate transcription factor nuclear factor erythroid 2-related factor 2 (Nrf2), leading to increased mRNA expression of Nrf2 target genes heme oxygenase-1 (HO-1). HO-1 further demonstrates protective function in cardiac injury induced by hypoxia/reoxygenation [9]. Nrf2/HO-1 signaling also demonstrates cytoprotective effects in LPS-induced cardiomyopathy [10].

The NLR family pyrin domain containing 3 (NLRP3) inflammasome is a mediator in the innate immune system. NLRP3 inflammasome includes three main members, namely NLRP3, apoptosis-associated speck-like protein containing a CARD (ASC), and procaspase-1. After activation, the NLRP3 inflammasome activates caspase-1, promotes the production of interleukin 18 (IL-18) and interleukin 1 beta (IL-1β), and consequently initiates inflammation [11]. NLRP3 inflammasome also contributes to cardiomyopathy in a sepsis model [12]. Moreover, inhibition of NLRP3 inflammasome mediates the cytoprotective effects in sepsis-associated myocardial injury [13, 14]. However, it remains unclear about the precise regulatory mechanisms of NLRP3 in septic cardiomyopathy.

Allicin is a volatile sulfide extracted from garlic bulbs and is responsible for most of the functions of garlic. Allicin demonstrates various physiological and pathophysiological effects, including anti-oxidant, antibacterial, anti-parasite activities [15]. Recently, Allicin has shown a variety of protective activities in cardiovascular disorders. For instance, Allicin reduced cardiomyocyte apoptosis and improved cardiac function in myocardial infarction rats [16]. Furthermore, Allicin also prevented myocardial apoptosis in diabetic rats and pathological overload-induced cardiac hypertrophy [17, 18]. However, it remains unclear about the effect of Allicin on myocardial injury induced by LPS. Therefore, in this study, we investigated the potential effects of Allicin on LPS‑induced cardiomyocyte injury and the underlying mechanisms.

## Materials and methods

### Cell culture

A rat embryonic-heart derived H9c2 cardiomyocyte cell line purchased from Shanghai Cell Bank of Chinese Academy of Sciences (Shanghai, China) was cultured in high-glucose Dulbecco’s Modified Eagle medium (DMEM, Gibco BRL, Gaithersburg, MD, USA) supplemented with 10% fetal bovine serum in a humidified incubator with 5% CO2 at 37 °C. The culture media were replaced every day, and when cell culture reaches 80–90% confluence, they were subcultured. Cells were pretreated with Allicin (25, 50, and 100 µM) for 2 h, followed by incubation with LPS (10 µg/mL) for 24 h at 37 °C.

### Cell transfection

H9c2 cells were seeded in 6-well plates and grew to about 80% confluence, and then incubated with a serum-free medium. The Nrf2 inhibitor (siNrf2) and negative control (siNC) were designed and synthesized by GenePharma (Shanghai, China). The sequence of Nrf2 siRNA is 5’-GAGGAUGGGAAACCUUACUTT-3′, and the sequence of siNC is 5’-CCAGAATGUAGUGTACGUGAU-3′. H9c2 cells were transfected with siNrf2 and siNC using Lipofectamine 3000 (Invitrogen). The efficiency of inhibition on Nrf2 was verified by western blot analysis at 48 h.

### Cell viability assay

Cell viability was measured by Cell Counting kit-8 (CCK-8; Sigma) according to manufactures instructions. Briefly, H9c2 cells were seeded in 96-well plates (1 × 10^4^ cells/well in 100 µL) and treated with different concentrations of Allicin (0, 25, 50, and 100 µM), followed by LPS (10 µg/ml). After 12, 24, and 36 h, cells were added with CCK-8 solution (10 µL) and incubated for 4 h at 37 °C. The absorbance was determined at 450 nm using a microplate reader (Bio-Rad Laboratories, CA, USA), and the values in each well were normalized to the control group.

### Lactate dehydrogenase (LDH) assay

Cell injury was evaluated by detecting the LDH release into the culture medium using an LDH assay kit (A020-3, Jiancheng, Nanjing, China) according to manufacturer instructions. Briefly, cells were treated with Allicin and LPS, 0.2mL culture medium was collected to determine the amount of LDH by spectrophotometry. All data are expressed as U/dL.

### TUNEL assay

H9c2 cells were fixed with 4% paraformaldehyde and permeabilized with 1% Triton X-100. Then cells were incubated with 50 µL TUNEL (Beyotime Institute of Biotechnology, Jiangsu, China) at 37 °C for 1 h. After washing twice with PBS, H9c2 cells were incubated with DAPI reaction mixture for 1 h at 37 °C to stain nuclei. Finally, cells were observed under a fluorescence microscope (515–565 nm; Olympus, BX71, Japan). TUNEL-positive cells were calculated from five photos (magnification ×200) for each sample.

### Caspase-3 activity assay

Cells were collected and incubated with lysis buffer for 15 min on ice, followed by centrifugation at 3000 g for 15 min to get supernatant. Then, 10 µL supernatants were mixed with 90 µL AC-DEVD-pNA substrate solution (Beyotime, Shanghai, China) at 37 °C for 1 h. Absorbance at 405 nm was determined using a microplate reader.

### Oxidative stress

The MDA content and SOD activity were measured in the cell lysate with a commercial assay kit (Nanjing Jiancheng Bioengineering Institute, Nanjing, China) according to manufacturer instructions. The MDA and SOD results were normalized to total protein and expressed as nmol/mg and U/mg, respectively.

### Enzyme-linked immunosorbent assay (ELISA)

ELISA was used to measure the levels of inflammatory cytokines in the culture medium of H9c2 cells. After various treatments, supernatants were collected and levels of interleukin-1β (IL-1β, RLB00), interleukin-6 (IL-6, R6000B), and tumor necrosis factor-alpha (TNF-α, RTA00) were measured by ELISA kits (R&D Systems Inc, Minneapolis, MN, USA). The concentrations were expressed as pg/mL.

### Real-time quantitative PCR (RT-qPCR)

Total RNA was extracted from H9c2 cells with TRIzol (Invitrogen) according to manufacturer instruction and used as templates (1 µg) to synthesize cDNA using a PrimeScript RT Reagent kit (TaKaRa Bio Inc, Dalian, China). RT-qPCR was carried out using an SYBR Green RT‑qPCR kit (Takara) on an Applied Biosystems 7500 real-time PCR system (Applied Biosystems, CA, USA). GAPDH was used as an internal reference. The PCR primers sequence were as follows: Nrf2 forward: 5′-ATT GCT GTC CAT CTC TGT CAG-3′; Nrf2 reverse: 5′-GCT ATT TTC CAT TCC CGA GTT AC-3′. HO-1 forward: 5′-GCC TGG CAC ATT TCC CTC AC-3′; HO-1 reverse: 5′-CAG AAC AGC CGC CTC TAC CG-3′. The RT-qPCR conditions were as following: denaturation at 95˚C for 7 min; followed by 40 cycles at 95˚C for 15 s and 60˚C for 1 min. Each measurement was performed in duplicate, and the relative mRNA level was calculated by the 2-ΔΔCt method [19].

### Western blot

Total protein was extracted using RIPA lysis buffer, and nuclear protein was extracted using Extraction Reagents (Pierce Biotechnology, Inc., Rockford, IL. USA). Protein concentrations were measured using a BCA protein assay kit (Beyotime Biotechnology, China). Protein samples (50 µg) were separated on 10% SDS-PAGE and transferred to PVDF membranes. Membranes were blocked with 5% low-fat milk, and incubated with primary antibodies Nrf2 (1:200, Cat# ab92946, Abcam, UK), HO-1 (1:500, Cat# ab13243, Abcam, UK), NLRP3 (1:500, Cat# ab214185, Abcam, UK), caspase-1 (1 : 200, Cat# sc-392,736, Santa Cruz, USA), and IL-1β (1 : 1000, Cat# ab9787, Abcam, UK). The membranes were incubated with HRP-linked secondary antibody. The β-actin (1:1000, Cat# ab8227, Abcam) was used as an internal control. The bands were visualized by ECL (Thermo, Waltham, MA, USA), and were analyzed using the ImageJ software.

### Statistical analysis

Data were expressed as mean ± standard deviation (SD), and analysis was carried out using SPSS 20.0 statistical software (IBM Analytics, New York, USA). The differences between three or more groups were statistically analyzed by one-way ANOVA. P < 0.05 was considered to indicate statistical significance.

## Results

### Allicin increased the viability of cardiomyocytes treated with LPS

**Fig. 1:**
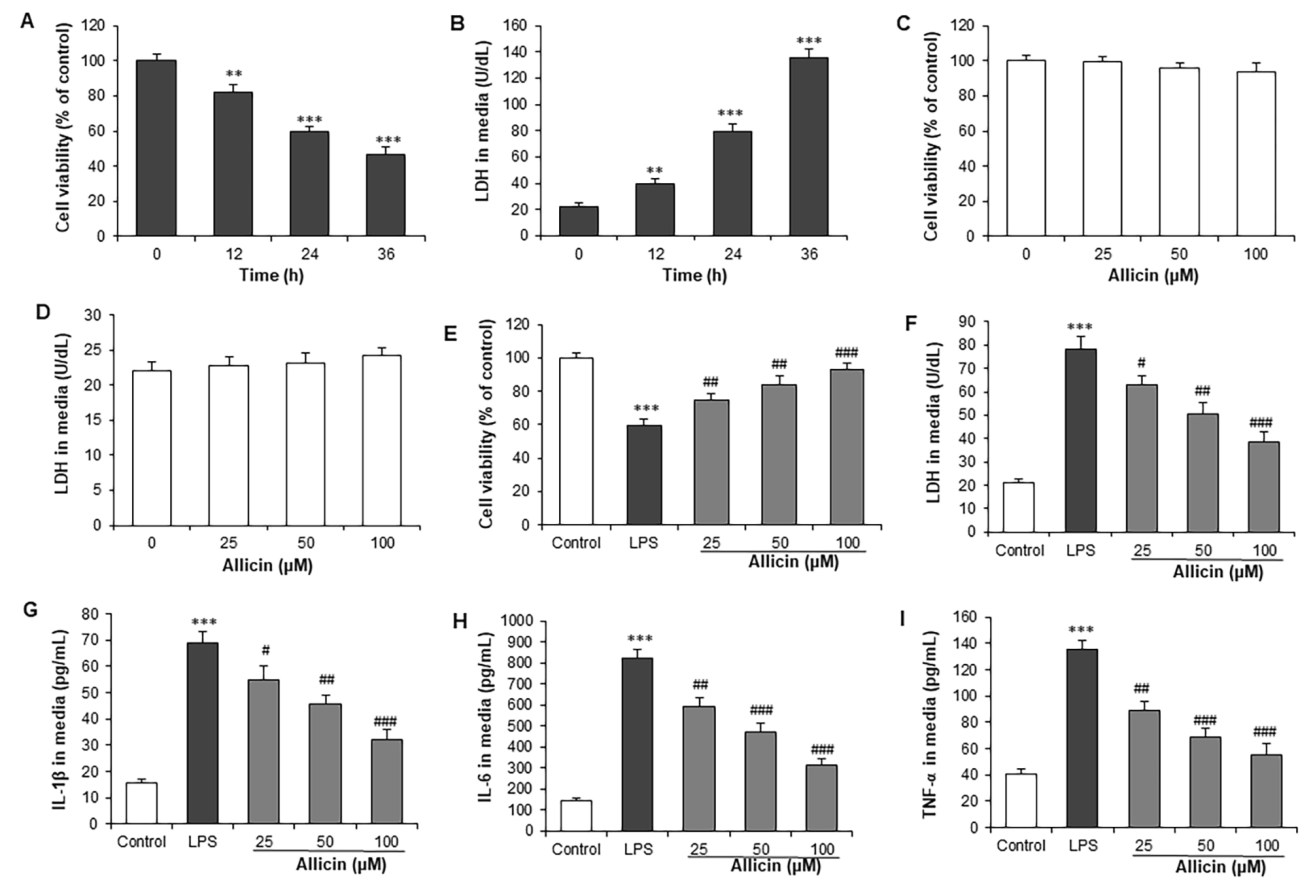
Allicin increases the viability of rat cardiomyocytes treated with LPS. H9c2 cells were treated with LPS (10 µg/mL) for 12, 24, and 36 h. **(A)** Cell viability was assessed by CCK-8 assay. **(B)** Cell injury was detected by LDH release assay. H9c2 cells were incubated with Allicin (25, 50, and 100 µM) for 24 h, **(C)** viability and **(D)** LDH content were measured. H9c2 cells were pretreated with Allicin (0, 25, 50, and 100 µM) for 2 h, followed by incubation with LPS (10 µg/mL) for a further 24 h. **(E)** CCK-8 assay and **(F)** LDH release assay was performed. ELISA assay was carried out to measure the concentrations of IL-1β **(G)**, IL-6 (H), and TNF-α **(I)** in culture media of H9c2 cells. ANOVA was performed to analyze the data. ***P < 0.001 vs. control group; #P < 0.05, ##P < 0.01, ###P < 0.001 vs. LPS group

H9c2 cells were treated with LPS (10 µg/mL) for 12, 24, and 36 h. Cytotoxicity was evaluated by CCK‑8 and LDH release assays. LPS treatment significantly reduced cell viability and increased LDH release in culture media at all time points (Fig. 1A and B). H9c2 cells were treated with different concentrations (0, 25, 50, and 100 µM) of Allicin for 24 h. There was no significant difference in the cell viability and supernatant LDH content of H9c2 cells between the Allicin group and the control group (Fig. 1C and D). The results indicated that Allicin shows no cytotoxicity on H9c2 cells. In LPS-stimulated H9c2 cells, Allicin pretreatment markedly increased the cell viability and decreased LDH content after 24 h treatment (P < 0.05) (Fig. 1E F). Furthermore, ELISA assay showed that LPS significantly increased the levels of IL-1β, IL-6, and TNF-α in H9c2 cells, but these increase in LPS-induced proinflammatory cytokine releases were reversed by co-treatment with Allicin (P < 0.05) (Fig. 1G H, 1I).

### Allicin attenuated LPS‑induced apoptosis and oxidative stress in cardiomyocytes

**Fig. 2:**
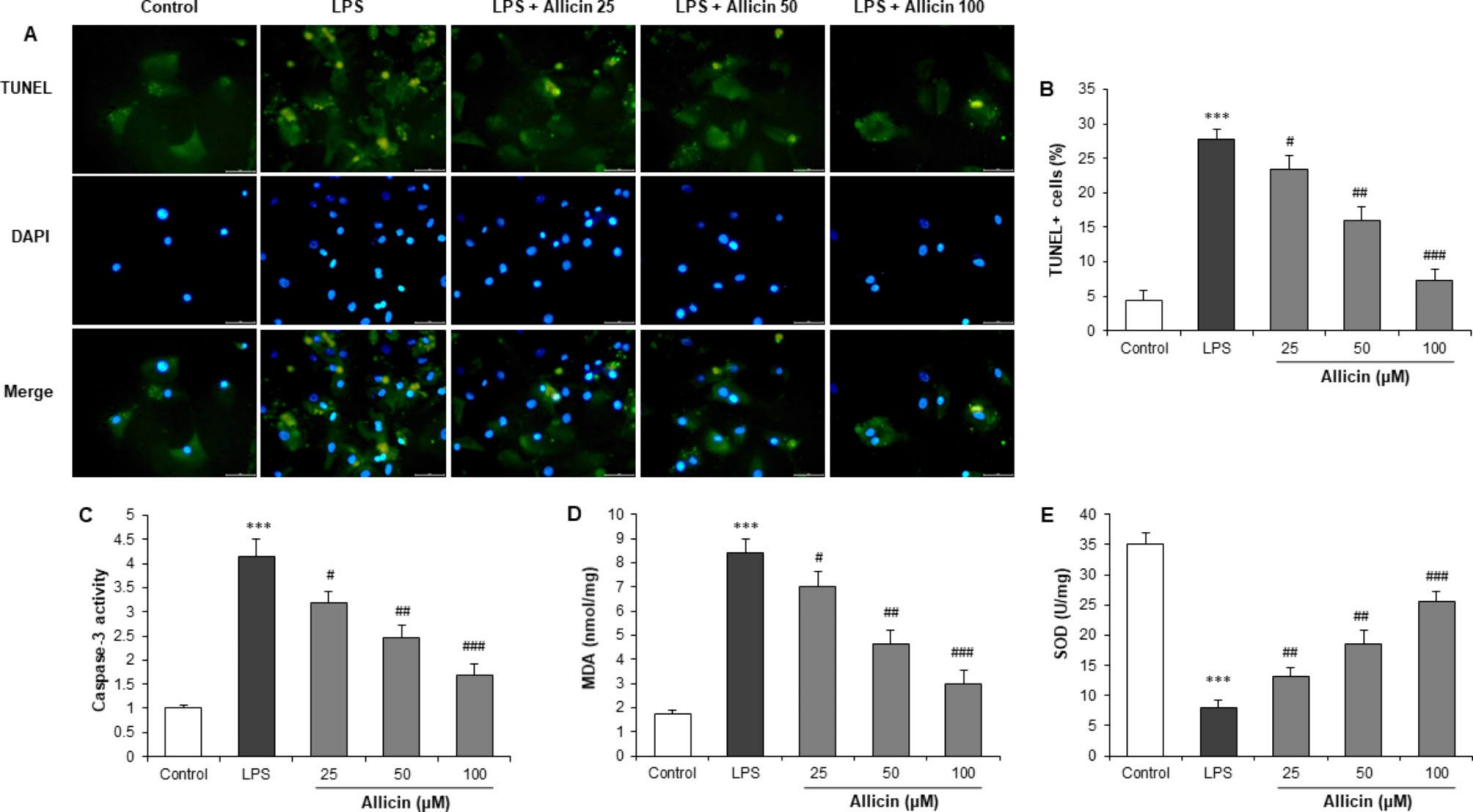
Effect of Allicin on cell apoptosis and oxidative stress in cardiomyocytes. H9c2 cells were pretreated with Allicin (0, 25, 50, and 100 µM) for 2 h, followed by incubation with LPS (10 µg/mL) for a further 24 h. **(A)** Representative TUNEL and DAPI‑stained images. Magnification, x200. **(B)** Quantitative analysis of TUNEL staining. **(C)** Cell apoptosis was determined by measurement of caspase-3 activity. The MDA level **(D)** and SOD activity **(E)** were measured by respective commercial kits. ***P < 0.001 vs. control group; #P < 0.05, ##P < 0.01, ###P < 0.001 vs. LPS group

H9c2 cells were stained with TUNEL to evaluate its effect on apoptosis. LPS stimulation markedly increased the number of TUNEL‑positive nuclei compared with control cells. However, the pretreatment of Allicin (25, 50, and 100 µM) gradually increased the attenuation of LPS‑induced cell apoptosis (Fig. 2A and B). Moreover, Allicin pretreatment also reduced caspase-3 activity in H9c2 cells with LPS induction (Fig. 2C). MDA and SOD are important biomarkers of oxidative stress and are widely used as indicators of oxidative injury. To examine the effect of Allicin on LPS-induced oxidative stress, the MDA level, and SOD activity were measured. Increased MDA content and decreased SOD activity were observed in H9c2 cells following LPS stimulation compared with control cells. However, pretreatment with Allicin restored the increase of MDA and decrease of SOD in LPS-stimulated H9c2 cells (Fig. 2D and E).

### Allicin activated Nrf2-HO-1 pathway in LPS-induced cardiomyocytes

**Fig. 3:**
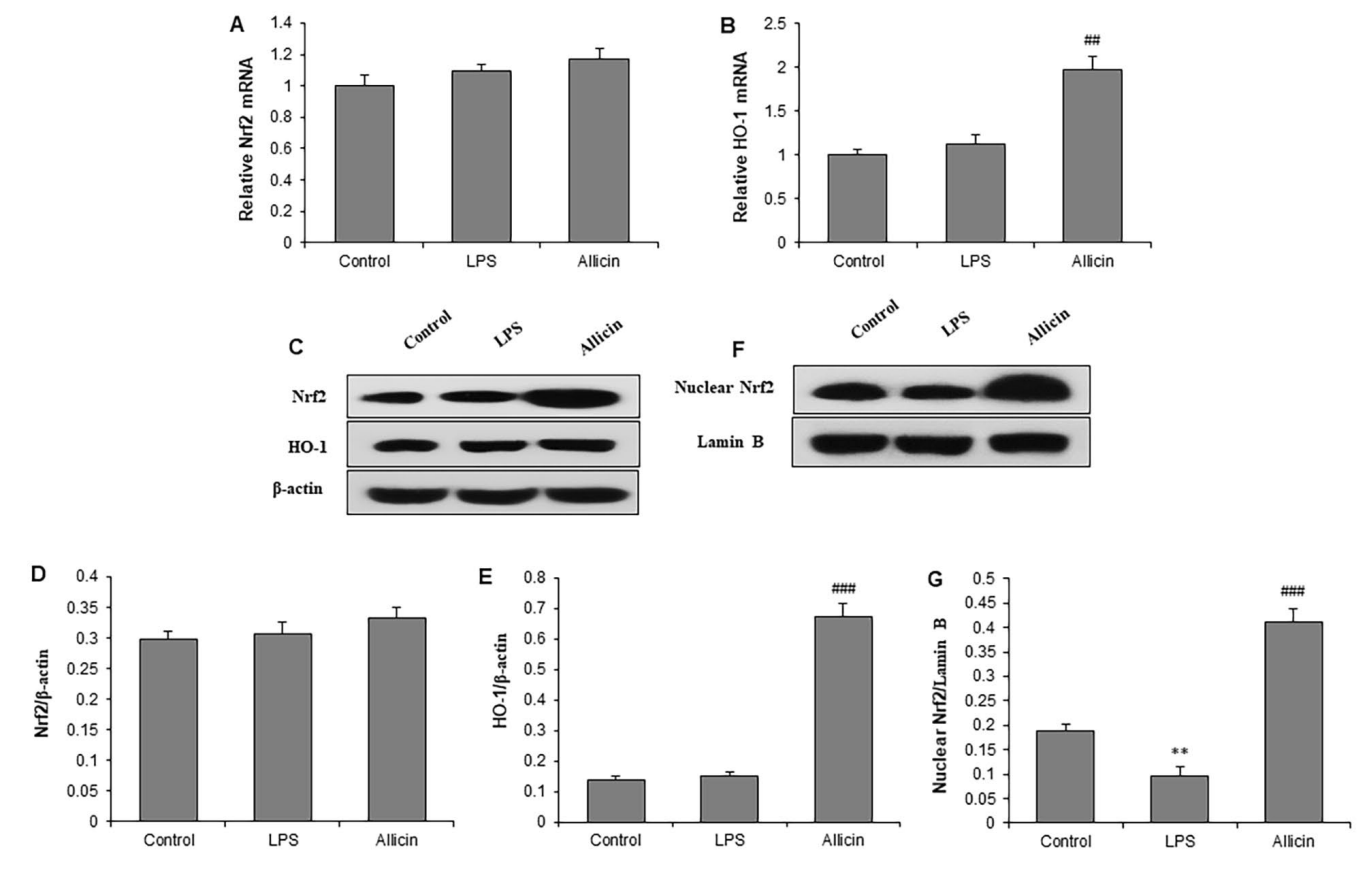
Allicin activated Nrf2-HO-1 signaling pathway in LPS-induced cardiomyocytes. H9c2 cells were pretreated with 50 µM Allicin, followed by incubation with LPS (10 µg/mL) for 24 h. RT-qPCR showed that Allicin did not change mRNA expression of Nrf2 **(A)** but enhanced mRNA expression of HO-1 **(B)** in H9c2 cells with LPS. **(C)** Representative western blot images investigating Nrf2 and HO-1. Allicin did not change Nrf2 protein **(D)** but enhanced HO-1 protein **(E)**. **(F)** Representative western blot images investigating nuclear Nrf2 protein. **(G)** Quantitative results of western blot analysis on nuclear Nrf2. **P < 0.01 vs. control group; ##P < 0.01, ###P < 0.001 vs. LPS group

To explore the molecular mechanism underlying suppressed oxidative stress by Allicin, we determined the mRNA and protein expressions of Nrf2 (total and nuclear Nrf2) and HO-1. RT-qPCR showed that Allicin did not change the mRNA expression of total Nrf2 but significantly increased the mRNA expressions of HO-1 in H9c2 cells after exposure to LPS (P < 0.05) (Fig. 3A and B). Western blot showed similar results of Allicin on protein expression of total Nrf2 and HO-1 (Fig. 3C, D and E). However, Allicin significantly increased the expression of nuclear Nrf2 protein in the nucleus of LPS-stimulated H9c2 cells (Fig. 3F and G).

### Allicin suppressed LPS-induced cardiomyocytes injury by NLRP3 inflammasome pathway

**Fig. 4:**
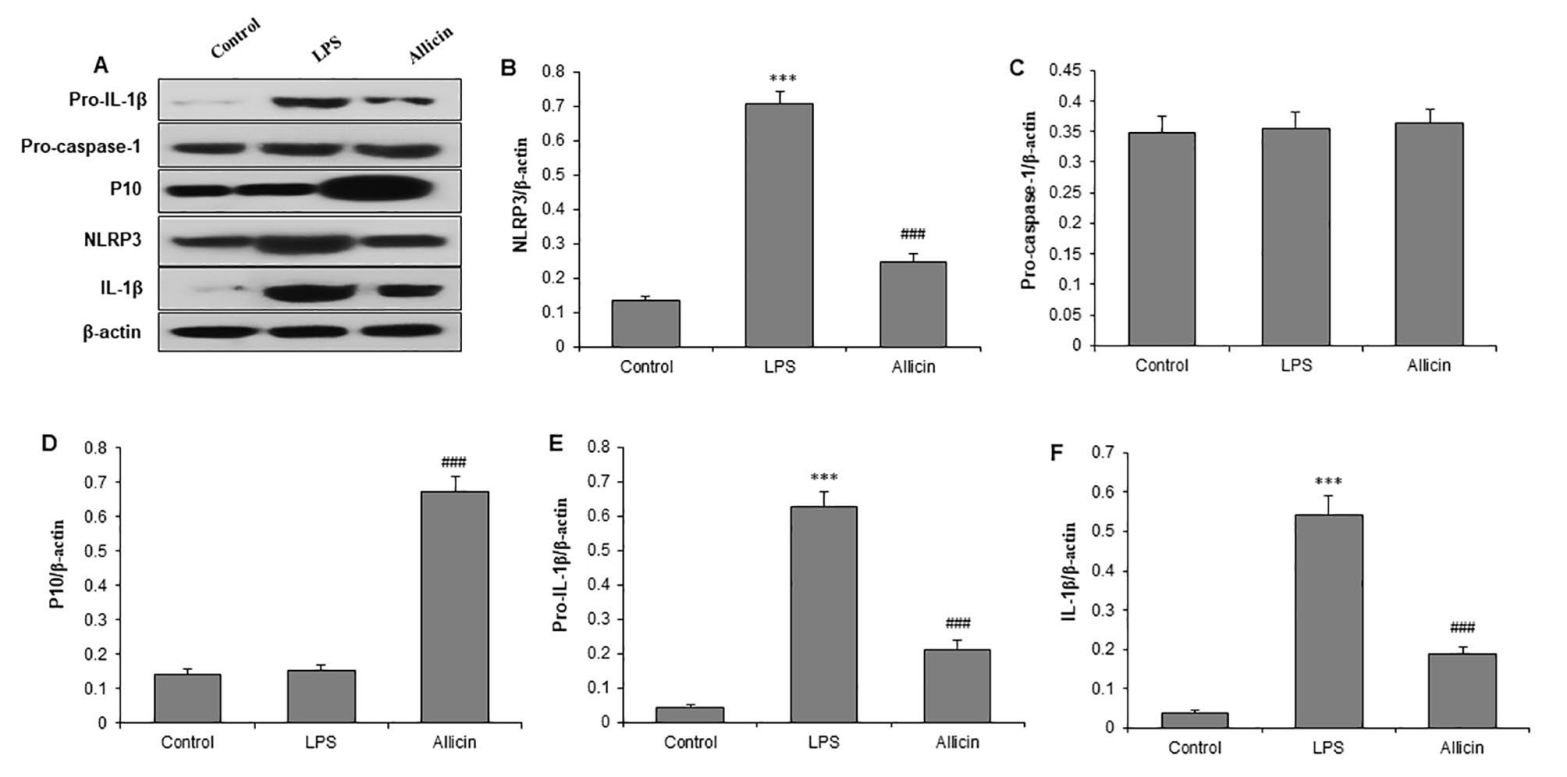
Allicin suppresses NLRP3 inflammasome activation by LPS in cardiomyocytes. H9c2 cells were pretreated with 50 µM Allicin, followed by incubation with LPS (10 µg/mL) for 24 h. **(A)** Representative western blot bands investigating NLRP3 pathway proteins. Quantitative results of western blot analysis on expressions of NLRP3 **(B)**, pro-caspase-1 **(C)**, cleaved caspase-1 (p10) protein **(D)**, pro-IL-1β **(E)**, and pro-IL-1β **(F)**. ***P < 0.001 vs. control group; ###P < 0.001 vs. LPS group

To investigate whether the NLRP3 inflammasome is involved in Allicin-suppressed H9C2 cell injury under LPS stimulation, we next measured protein levels of NLRP3 related proteins (Fig. 4A). LPS could activate the NLRP3 inflammasome characterized by increasing the protein level of NLRP3 compared with that of LPS groups, which was reversed by Allicin pretreatment (Fig. 4B). Compared with H9c2 cells with LPS, Allicin pretreatment did not change the pro-caspase-1 protein but increased the expression of cleaved caspase-1 (p10) protein in H9c2 cells (Fig. 4C and D). Moreover, Allicin significantly reversed the LPS-stimulated increase in pro-IL-1β and IL-1β protein in H9c2 cells (Fig. 4E F). These results indicate that Allicin pretreatment suppresses NLRP3 inflammasome and inflammatory pathway, which are activated by LPS in H9c2 cells.

### Nrf2 mediated the protection against injury and inhibition of NLRP3 by Allicin

**Fig. 5:**
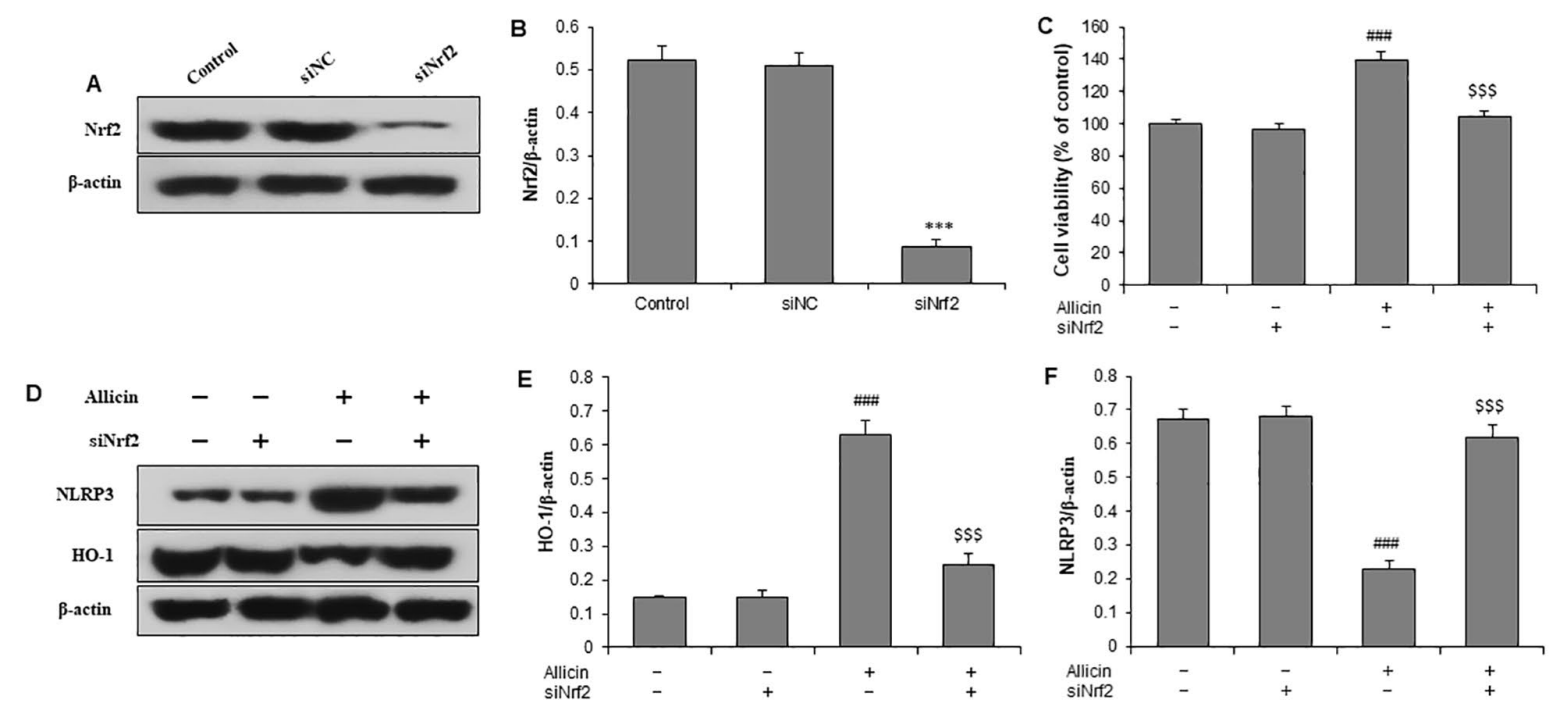
Nrf2 mediates the protective effects of Allicin on LPS-induced injury and NLRP3 inhibition. H9c2 cells were transfected with Nrf2 siRNA, and the silencing efficiency was verified by western blot at 48 h after transfection **(A, B)**. **(C)** Cell viability of H9c2 cells with Nrf2 silencing. **(D)** Representative western blot images of HO-1 and NLRP3. **(E-F)** Quantitative results show that Allicin increased HO-1 protein and decreased NLRP3 protein, which was reversed by silencing of Nrf2 (siNrf2). ***P < 0.001 vs. control group; ###P < 0.001 vs. LPS group; $$$P < 0.001 vs. Allicin group

We then carried out siRNA to specifically targeting Nrf2 in cardiomyocytes. The transfection efficiency and silencing effect were validated by western blot, with an inhibitory rate of about 85% (Fig. 5A and B). Knockdown of Nrf2 significantly increased the cell viability in H9c2 cells after Allicin pretreatment (Fig. 5C). Furthermore, compared to H9c2 cells with Allicin, knockdown of Nrf2 significantly decreased HO-1 protein and increased NLRP3 protein expressions (Fig. 5D and E F). These results suggest that Allicin plays a protective effect on LPS-induced injury of cardiomyocytes by Nrf2-HO-1-NLRP3 pathway.

## Discussion

In this study, we investigated the effect of Allicin in H9c2 cell lines treated with LPS. Allicin showed no toxicity on H9c2 cells. However, Allicin attenuated LPS-induced injury of H9c2 cells, as evidenced by improved cell viability, and reduced LDH and inflammatory cytokine release, apoptosis, and oxidative stress. The protective effects of Allicin might be through activation of Nrf2-HO-1 pathway and inhibition of NLRP3 inflammasome. Therefore, Allicin could be a potential therapeutic agent for the prevention and treatment of septic cardiomyopathy.

Allicin has shown various beneficial effects on cardiovascular diseases (such as atherosclerosis and stroke) [16, 20, 21]. Moreover, Allicin protected against hypertrophy and apoptosis in cardiomyocytes through reducing oxidative stress [22]. Most importantly, Allicin decreases suppressed oxidative stress and inflammation in LPS-induced endothelial cells through activation of Nrf2 [23]. We demonstrated that Allicin can also have a protective effect on LPS-induced cardiomyocytes and is a possible treatment drug for septic cardiomyopathy because endothelial cells and cardiomyocytes share the same origin. We then administered Allicin to H9c2 cell lines after LPS incubation, and the results revealed that Allicin boosted cell viability and decreased apoptosis. The mechanisms of Allicin could be associated with activation of the Nrf2-HO-1 pathway and inhibition of NLRP3 inflammasome.

One major pathophysiology of septic cardiomyopathy is excessive reactive oxygen species (ROS) and reduced antioxidants [8]. MDA is an indicator of oxidative damage and SOD is an important myocardial antioxidant enzyme. In this study, Allicin attenuated LPS-induced increase of MDA content and decrease in SOD activity. These oxidative inhibitions by Allicin were also reported in LPS-induced H9c2 cell lines treated by other cardioprotective agents [24]. Our study also showed translocation of Nrf2 protein to the nucleus and enhanced HO-1 expression by Allicin in LPS-induced H9c2 cell lines. Moreover, the protective effect of Allicin was abolished by silencing of Nrf2. Therefore, Allicin could be protected against myocardial damage caused by LPS, and the mechanism may be connected to anti-oxidative activity via activation of the Nrf2/HO-1 pathway.

One principle mechanism in LPS-induced myocardial injury is an overproduction of inflammatory mediators, including IL‑1β, IL‑6, and TNF‑α, which are mainly released from cardiomyocytes [25]. These inflammatory mediators further influence downstream pathways and aggravate the myocardial injury, apoptosis, and heart failure induced by LPS [26, 27]. Therefore, inhibition of the inflammatory pathway may act as a therapeutic strategy in combating cardiac injury. Allicin has been shown to suppress the release of IL-1β, IL-6, and TNF-α in the serum of septic lung injury and doxorubicin-induced cardiotoxicity rats [28, 29]. Our study showed similar results in LPS-induced H9c2 cell lines and indicates an anti‑inflammatory effect of Allicin in cardiomyocytes.

NLRP3 activation leads to the formation of NLRP3 inflammasome, and Caspase-1 promotes the release of inflammatory cytokines, such as IL-1β [11]. Sepsis triggers NLRP3 activation in cardiomyocytes and cardiac fibroblasts [12, 30]. The present study showed that Allicin reduced cleaved caspase-1 (p10), pro-IL-1β, and IL-1β protein in H9c2 cell lines with LPS. NLRP3 inflammasome is regarded as a target of septic cardiomyopathy and its inhibition mediated the cytoprotective effects and improved myocardial function in sepsis-associated myocardial injury [13, 14]. A recent work found that Allicin reduces NLRP3 inflammasome in the mouse hippocampus of depressive-like behaviors supports the addition of Allicin to the list of drugs that target NLRP3 inflammasome in septic cardiomyopathy in our study [31]. NLRP3 inflammasome can promote cardiomyocyte remodeling and aggravate cardiac fibrosis and hypertrophy [32, 33]. Therefore, it can be speculated that Allicin might be also an inhibitor of NLRP3 inflammasome in cardiac injury and hypertrophy induced by diabetes and pathological overload [17, 18], which needs further investigation.

Our study demonstrated the involvement of the Nrf2-HO-1-NLRP3 pathway in LPS-induced cardiac damage, as demonstrated by Allicin’s mitigation of the decrease in the NLRP3 pathway protein. ROS is an important activator of the NLRP3 inflammasome [34]. The ROS-dependent NLRP3 inflammasome activation was also observed in LPS-induced cardiomyocyte injury and sepsis‑induced brain injury [35, 36]. Therefore, as an important antioxidative transcription factor, Nrf2 is a potential activator of NLRP3 inflammasome in various pathological conditions, such as depressive-like behaviors, cerebral ischemia-reperfusion injury, and diabetic nephropathy [37–39]. The Nrf2-NLRP3 pathway has only ever been associated with cardiac ischemia-reperfusion damage, hence it is currently unknown if it is also active in other types of cardiomyopathy and needs additional research.

In summary, Allicin can protect H9c2 cardiomyocytes from LPS-induced injury, and increased cell viability and survival suppress the production of inflammatory mediators. The cardioprotective effects of Allicin may be due to activation of Nrf2-HO-1 and inhibition of NLRP3 inflammasome. Allicin might be a potential agent for the prevention and treatment of septic cardiomyopathy. Further study still needs to confirm its efficacy in vivo and explore more detailed mechanisms.

### Electronic supplementary material

Below is the link to the electronic supplementary material.

Supplementary Information: Representative original western blot images

## Data Availability

The datasets used and/or analyzed during the current study are available from the corresponding author on reasonable request.
