# Supplementary material for: Allicin protects against LPS-induced cardiomyocyte injury by activating Nrf2-HO-1 and inhibiting NLRP3 pathways
Source: BMC Cardiovasc Disord. 2023 Aug 18;23:410. doi: 10.1186/s12872-023-03442-1 (PMC10439633; doi:10.1186/s12872-023-03442-1)
Supplement: Supplementary file 1 — Supplementary Information: Representative original western blot images [file 12872_2023_3442_MOESM1_ESM.ppt]

## Slide 1
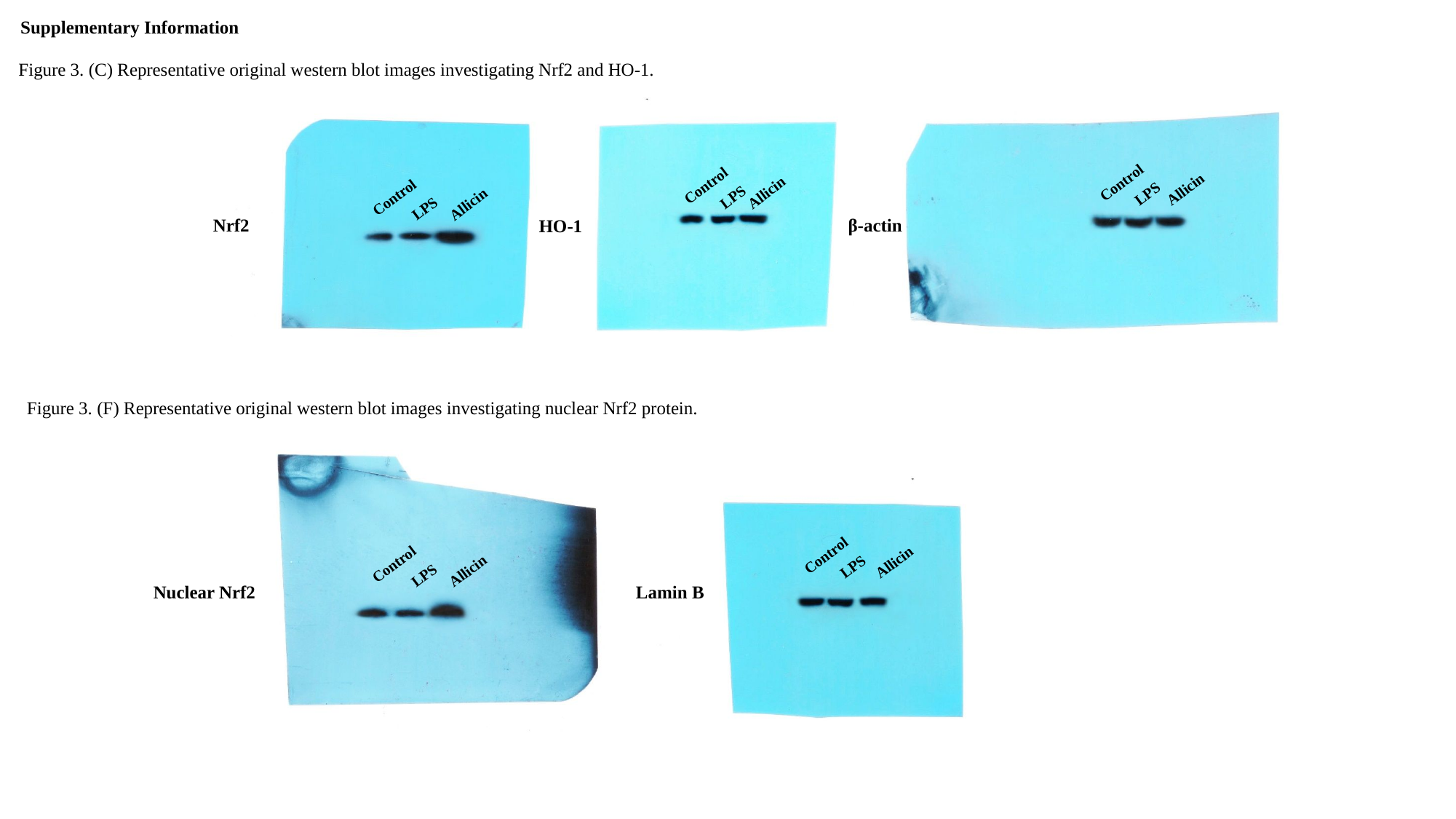

Supplementary Information
Figure 3. (C) Representative original western blot images investigating Nrf2 and HO-1.
Control
Control
Allicin
Allicin
LPS
Control
LPS
Allicin
LPS
Nrf2
β-actin
HO-1
Figure 3. (F) Representative original western blot images investigating nuclear Nrf2 protein.
Control
Allicin
Control
LPS
Allicin
LPS
Lamin B
Nuclear Nrf2

## Slide 2
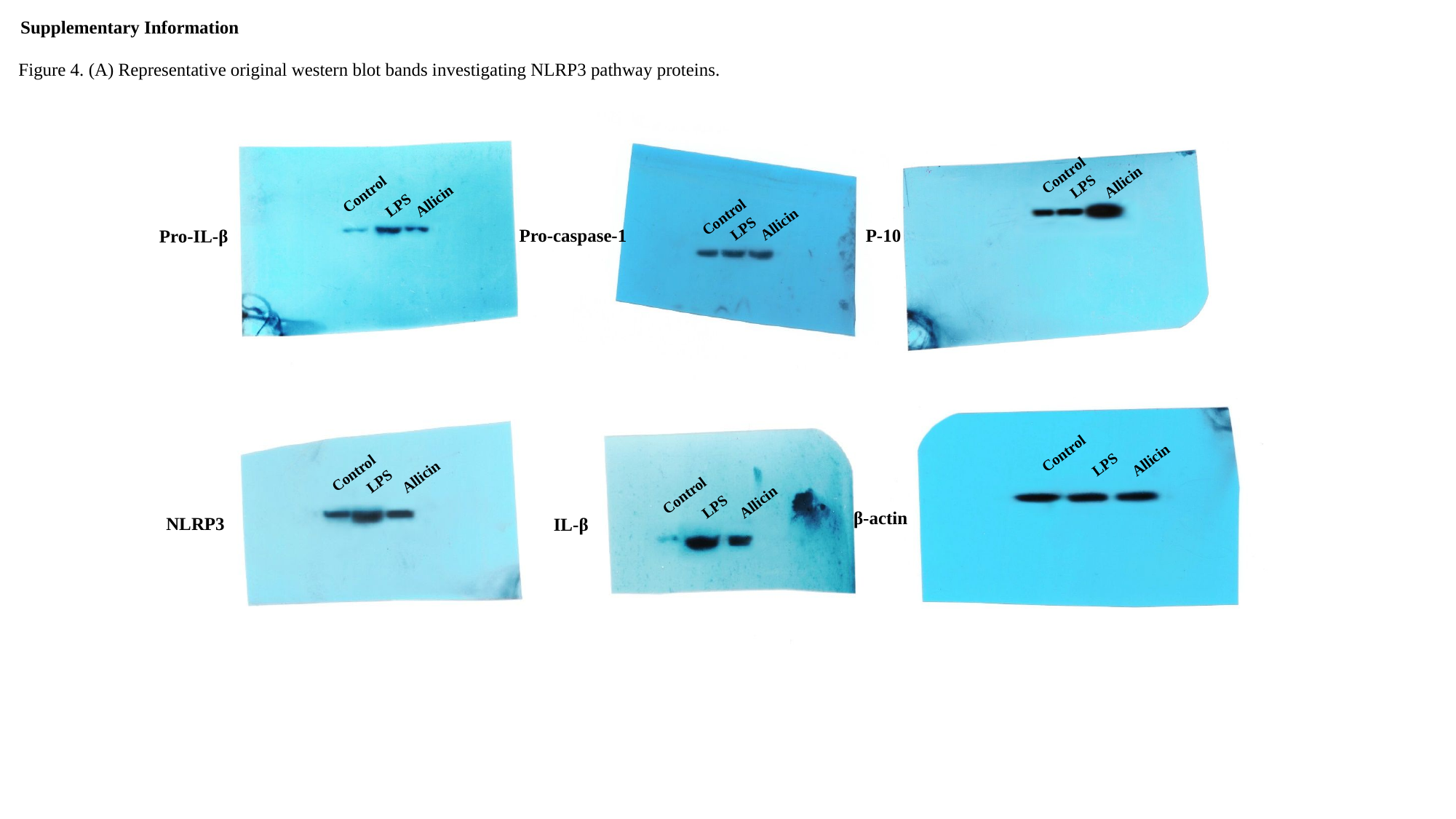

Supplementary Information
Figure 4. (A) Representative original western blot bands investigating NLRP3 pathway proteins.
Control
Allicin
LPS
Control
Allicin
LPS
Control
Allicin
LPS
Pro-caspase-1
P-10
Pro-IL-β
Control
Allicin
LPS
Control
Allicin
LPS
Control
Allicin
LPS
β-actin
NLRP3
IL-β

## Slide 3
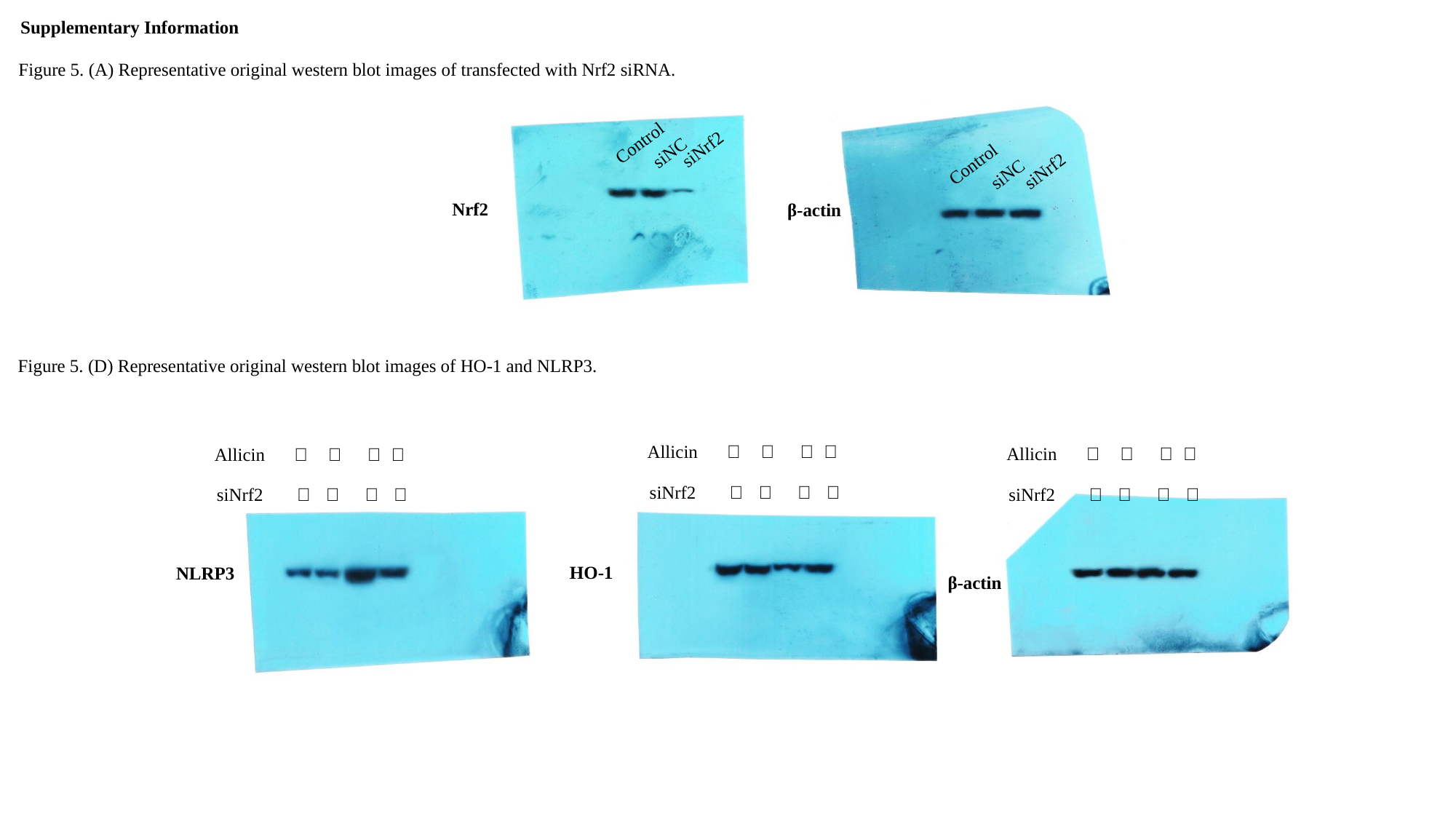

Supplementary Information
Figure 5. (A) Representative original western blot images of transfected with Nrf2 siRNA.
Control
siNrf2
 siNC
Control
siNrf2
 siNC
Nrf2
β-actin
Figure 5. (D) Representative original western blot images of HO-1 and NLRP3.
Allicin
➖ ➖ ➕ ➕
siNrf2
➖ ➕ ➖ ➕
Allicin
➖ ➖ ➕ ➕
siNrf2
➖ ➕ ➖ ➕
Allicin
➖ ➖ ➕ ➕
siNrf2
➖ ➕ ➖ ➕
HO-1
NLRP3
β-actin
